# Condomless Vaginal Intercourse and Its Associates among Men Who Have Sex with Men in China

**DOI:** 10.1371/journal.pone.0154132

**Published:** 2016-04-26

**Authors:** Hongcheng Shen, Songyuan Tang, Tanmay Mahapatra, Joseph D. Tucker, Shujie Huang, Bin Yang, Jinkou Zhao, Roger Detels, Weiming Tang

**Affiliations:** 1 Guangdong Provincial Center for Skin Disease and STI Control, Guangzhou, China; 2 University of North Carolina, Project-China, Guangzhou, China; 3 Department of Epidemiology, School of Public Health, University of California Los Angeles, Los Angeles, California, United States of America; 4 Monitoring and Evaluation Unit, the Global Fund to Fight AIDS, Tuberculosis and Malaria, Geneva, Switzerland; New England Biolabs, Inc., UNITED STATES

## Abstract

**Background:**

HIV prevalence has increased rapidly among men who have sex with men (MSM) in China reaching alarmingly high levels in some cities. Bisexual MSM have potential to transmit HIV and syphilis to their female partners through condomless vaginal intercourse (CVI). Thus, estimation of the burden of CVI and identification of its associates seemed necessary to control this cross-gender transmission.

**Method:**

In a cross-sectional study, using respondent-driven-sampling and snowball sampling, 2958 MSM were recruited from seven Chinese cities, interviewed and tested for HIV and syphilis. Descriptive analysis of the socio-demographic and behaviors followed by simple and multiple logistic regressions [adjusted for income, city, race and social network size to determine adjusted odds ratio (aOR)] were performed using SAS-9.1.

**Results:**

Among participating MSM, 19.03% were engaged in CVI. Prevalence of HIV and syphilis among participants involved in CVI were 5.86% and 14.74% respectively. MSM who were older [aOR for aged 40–49 = 2.60 (95% CI: 1.54–4.37)], married [aOR = 6.13 (4.95–7.58)], attended primary school or below [aOR = 3.86 (2.26–6.69)], met male partners at spa/bathhouse/sauna/massage parlor [aOR = 3.52 (2.62–4.72)] and had heterosexual orientation [aOR = 13.81 (7.14–26.70)] were more likely to have CVI. Furthermore, correct knowledge regarding HIV [aOR = 0.70 (0.55, 0.88)] and exposure to HIV prevention interventions [aOR = 0.67 (0.54, 0.82)] were negatively associated with CVI.

**Conclusions:**

CVI was found to be common among MSM in China. To minimize the transmission of HIV and syphilis from bisexual MSM to their relatively female partners, targeted interventions should specifically focus on bisexual MSM especially the older and married subgroups.

## Introduction

Worldwide it has been observed that Men who have Sex with Men (MSM) are quite vulnerable for acquisition of HIV. In all the global geographical regions, median HIV prevalence has crossed 1% among MSM [1]. Moreover, in multiple regions of the world, like in north, south and central America, south and south-east Asia and sub-Saharan Africa, HIV prevalence among MSM has become as high as 14–18% [2]. HIV epidemic among MSM in China is currently being considered as one of the toughest public health challenges that the country is facing [2]. According to the National Sentinel Surveillance data, over the years among MSM in China, HIV prevalence did show a rising trend from 0.9% in 2003 to 6.3% in 2011 while syphilis sero-positivity increased from 2.4% in 2004 to 7.8% in 2011[3].

There are several factors including multiple male sex partners, large network size [3–5] and high proportion of sexual intercourses without condom [6], those increase the potential risk for being infected with HIV and other sexually transmitted diseases (STDs) like syphilis among MSM in China. In the context of the Chinese society, the scenario becomes further complicated for this population resulting from the social pressure on males to meet the perceived masculine responsibility of becoming a father and known high risks for acquisition of HIV and other STDs among MSM [7, 8]. Thus, to comply with the social demand, many MSM marry women, engage in heterosexual activities with them and give birth to babies to conceal their homosexuality and fulfill their social and family responsibilities. This scenario was evident from the data that the proportion of heterosexual marriages among MSM in China has increased from 19.3% in 2008 to 27.9% in 2011[3]. Under this circumstance, Chinese MSM have become a crucial bridge population for the transmission of HIV/STDs from their male partners to their relatively heterosexual female partners through condomless sexual contacts, and to their children through vertical transmission. This hidden cross-gender bridging behavior often results in a relatively non-traceable spread of HIV/STDs, increasing the challenges for the HIV/STD control programs in this country.

Bisexual subgroup of MSM population thus required special attention in terms of the coverage of the interventions for the testing and prevention of HIV and other STDs. However, mostly due to their hidden nature, majority of studies involving MSM were focused on HIV/STD transmission between men and men, while little attention was paid to their potential bridging role in transmission of HIV to general female population [9–11]. Further, to the best of our knowledge, few studies did ever focus upon the associates of condomless vaginal intercourse (CVI) among MSM globally as well as in China, while identification of the factors associated with CVI in this population seemed critical for controlling the epidemics of HIV and other STDs.

To address this dearth of information and in order to help public health administrators in designing appropriate HIV prevention strategies, we used the data from a multi-centre cross-sectional study to determine the associates of CVI among Chinese MSM.

## Materials and Methods

The current analysis was conducted using cross-sectional data from seven Chinese cities, between 2008 and 2009. The detailed information regarding the data collection has been reported elsewhere [12]. In brief, respondent-driven-sampling (RDS) method (in Nanjing, Chongqing, Jinan, Harbin and Guangzhou) and snowball sampling (in Suzhou and Changzhou) were used to recruit eligible participants.

### Study sample

Males aged 18 years or more, who had oral and/or anal sex with men during last 12 months and were carrying valid reference coupons provided by other MSM from their social network, were eligible for the study if they were willing to provide biological sample for HIV and syphilis testing. Signed written informed consent was collected from each eligible participant, followed by counseling, blood specimen collection (for HIV and active syphilis testing) and interview.

### Data collection

Face-to-face interviews were conducted by trained personnel from Centers for Diseases Prevention and Control (CDC China) maintaining privacy using structured questionnaires.

#### Demographic and socio-economic measures

Demographic and socio-economic information on year of birth (age), education level, monthly income, ethnicity, marital status and city of residence were collected from each participant. Age (in years) was measured as continuous, and was further categorized into different age groups (<20; 20–29; 30–39; 40–49; 50 and above). Education was measured by highest level of education obtained and categorized into primary school or below, junior/senior high school, and college or above. Ethnicity was categorized into: Han and others (as Chinese population is predominantly Han). City of residence was classified as: cities under study, province of the cities under study and other provinces. Marital status was categorized into: ever married and never married. Monthly income was categorized into six groups (in RMB): No income; 1–1000; 1001–2000; 2001–3000; 3001–4000; and more than 4000. Participants providing correct answers to at least 6 of the 8 HIV related knowledge questions were considered to have correct knowledge of HIV transmission and prevention.

#### Risk behaviors

Following risk behavioral characteristics were assessed in the current study: venue of meeting partners, self-perceived sexual orientation, condom use during last anal sex, reported condomless anal intercourse (CAI) in past six months, symptoms of STD in past year, and reported condomless vaginal intercourse (CVI) in the last six months. Self-perceived sexual orientation was defined as the sexual role and predilection as perceived by the respondent himself and not the actual behavior he was engaged in. CAI was defined as never or only occasionally using condom during anal intercourse in the past six months with all male partners. CVI was defined as never or only occasionally using condom during vaginal intercourse with women in the last six months.

After the interview, each participant was reimbursed with approximately five US dollars for his travel and time contribution for the study. Further, a sum of approximately US $ 1.6 (per new recruit) was paid to participants who successfully recruited other MSM from their social networks. Study procedures were carried at local STD clinics run by provincial CDCs.

#### Serologic measures

Before the interview, five ml of venous blood was collected from each participant for HIV and syphilis testing. HIV antibodies were screened using a rapid test (Acon Biotech Co., Ltd, lot 200803973/WB). Screened positive samples were further confirmed by Western blot (HIVBLOT 2.2, Genelabs Diagnostics, Singapore, lot AE8039). Syphilis antibodies were screened using Rapid Plasma Reagin (RPR; Beijing WanTai Biological Pharmacy Enterprise Co., Ltd., lot N20080404) test and those individuals with a positive RPR titer were subjected to the Treponema Pallidum Particle Agglutination assay (TPPA; Livzon Group Reagent Factory, Guangdong, China, lot VN80803). Syphilis positivity was deemed as “current” when both TPPA and RPR assays were positive.

### Data analysis

Data were double-entered using the software EpiData 3.0 by the staffs of local CDCs. After the collection of data from all CDCs further logic checks were performed centrally. Identified errors were corrected by communicating with the local CDC and re-checking the original questionnaires. SAS version 9.1 was used for all statistical analyses. Descriptive analyses were conducted to determine the distribution of the demographic factors, behaviors, HIV and syphilis prevalence and other related information for both participants who were engaged in CVI and not. To identify the predictors of CVI among the participants, simple bivariate logistic regression analyses [Odds ratios (OR) and corresponding 95% confidence intervals (CI)] were conducted. To control for potential confounders, multiple logistic regressions were further performed to determine adjusted ORs (aOR) and corresponding 95% CIs. Sampling city (seven cities), annual income, social network size and race were included in these multiple logistic regression models (S1 Dataset).

### Ethics Statement

Signed informed consent was obtained from each of the participants prior to interview, blood collection and intervention. Each of the participants was free to withdraw from this survey at any time after recruitment without any consequence. The questionnaires and written consent documents were separately kept in locked cupboards at the study sites, and unauthorized persons were unable to access them. The study protocol, procedures and contents were reviewed and approved by the Ethics Committee of the China CDC in Beijing [13].

## Results

### Demographic characteristics

Among 2958 recruited MSM, about half (47.68%) had college or above level of education. More than half (57.44%) of participants belonged to 20–29 years age group and were (51.54%) residents of the cities under study. Majority of the participants belonged to the Han ethnicity (97.57%) and were never married (73.09%). In addition, about one-sixth (15.83%) of participants self-reported that they had no income, while 7.47% participants had monthly income more than 4000 RMB. About 68.56% of the participants received HIV related service in the last year while 79.55% subjects were found to have correct HIV related knowledge (Table 1).

**Table 1:** Distribution of socio-demographics, sexual behavior and HIV/syphilis prevalence among men who have sex with men in China, 2008–2009 (N = 2958).

	CVI(n = 563)			Non-CVI(n = 2395)			Total(n = 2958)	
Variables	Frequency	Percent	95% CI	Frequency	Percent	95% CI	Total	Percentage
Age								
*<20*	25	4.44	2.73,6.15	153	6.39	5.41,7.37	178	6.02
*20–29*	200	35.52	31.56,39.49	1499	62.59	60.65,64.53	1699	57.44
*30–39*	177	31.44	27.59,35.29	431	18	16.46,19.54	608	20.55
*40–49*	113	20.07	16.75,23.39	180	7.52	6.46,8.57	293	9.91
*50 and above*	48	8.53	6.21,10.84	132	5.51	4.60,6.43	180	6.09
Marital Status								
*Never Married*	205	36.41	32.43,40.40	1957	81.71	80.16,83.26	2162	73.09
*Ever Married*	358	63.59	59.60,67.57	438	18.29	16.74,19.84	796	26.91
Residency								
*City under study*	267	47.42	43.29,51.56	1257	52.51	50.50,54.51	1524	51.54
*The province of the city under study*	154	27.35	23.66,31.05	536	22.39	20.72,24.06	690	23.33
*Other provinces*	142	25.22	21.62,28.82	601	25.1	23.37,26.84	743	25.13
Race								
*Han*	556	98.76	97.84,99.67	2329	97.28	96.63,97.94	2885	97.57
*Others*	7	1.24	0.33,2.16	65	2.72	2.06,3.37	72	2.43
Education								
*Primary School or below*	26	4.62	2.88,6.36	42	1.75	1.23,2.28	68	2.3
*Junior or Senior High school*	385	68.38	64.53,72.24	1094	45.7	43.70,47.69	1479	50.02
*College or above*	152	27	23.32,30.68	1258	52.55	50.55,54.55	1410	47.68
Monthly income								
*No income*	40	7.1	4.98,9.23	428	17.88	16.34,19.41	468	15.83
*1 to 1000*	54	9.59	7.15,12.03	417	17.42	15.90,18.94	471	15.93
*1001 to 2000*	221	39.25	35.21,43.30	715	29.87	28.03,31.70	936	31.65
*2001 to 3000*	141	25.04	21.45,28.63	419	17.5	15.98,19.03	560	18.94
*3001 to 4000*	64	11.37	8.74,14.00	237	9.9	8.70,11.10	301	10.18
*More than 4000*	43	7.64	5.44,9.84	178	7.44	6.38,8.49	221	7.47
Venue								
*Pub*, *Disco*, *Tearoom*, *or Club*	115	20.43	17.09,23.77	321	13.41	12.04,14.77	436	14.74
*Spa or bathhouse*, *Sauna or Massage*	126	22.38	18.93,25.83	235	9.82	8.62,11.01	361	12.21
*Public Restroom*, *or Public Lawn*	100	17.76	14.60,20.93	223	9.32	8.15,10.48	323	10.92
*Internet*	164	29.13	25.37,32.89	1446	60.4	58.44,62.36	1610	54.45
*Others*	58	10.3	7.78,12.82	169	7.06	6.03,8.09	227	7.68
Sexual orientation								
*Homosexual*	109	19.36	16.09,22.63	1517	63.37	61.44,65.30	1626	54.99
*Heterosexual*	23	4.09	2.45,5.73	20	0.84	0.47,1.20	43	1.45
*Bisexual*	394	69.98	66.18,73.78	736	30.74	28.89,32.59	1130	38.21
*Not sure*	37	6.57	4.52,8.63	121	5.05	4.18,5.93	158	5.34
Social network size								
*2 or less*	118	20.96	17.59,24.33	266	11.11	9.85,12.37	384	12.98
*3 to 9*	245	43.52	39.41,47.62	891	37.2	35.27,39.14	1136	38.4
*10 and above*	200	35.52	31.56,39.49	1238	51.69	49.69,53.69	1438	48.61
Knowledge								
*No*	138	24.51	20.95,28.08	467	19.5	17.91.21.09	605	20.45
*Yes*	425	75.49	71.92,79.05	1928	80.5	78.91,82.09	2353	79.55
Coverage								
*No*	226	40.14	36.08,44.20	704	29.39	27.57,31.22	930	31.44
*Yes*	337	59.86	55.80,63.92	1691	70.61	68.78,72.43	2028	68.56
City								
*Nanjing*	74	13.14	10.34,15.94	357	14.91	13.48,16.33	431	14.57
*Suzhou*	86	15.28	12.29,18.26	194	8.1	7.01,9.19	280	9.47
*Yangzhou*	115	20.43	17.09,23.77	185	7.72	6.65,8.79	300	10.14
*Chongqing*	58	10.3	7.78,12.82	559	23.34	21.65,25.04	617	20.86
*Guangzhou*	47	8.35	6.06,10.64	332	13.86	12.48,15.25	379	12.81
*Ha'erbin*	67	11.9	9.22,14.58	384	16.03	14.56,17.50	451	15.25
*Ji'nan*	116	20.6	17.25,23.96	384	16.03	14.56,17.50	500	16.9
CAI								
*Yes*	295	52.4	48.26,56.54	1146	47.85	45.85,49.85	1441	48.72
*No*	268	47.6	43.46,51.74	1249	52.15	50.15,54.15	1517	51.28
HIV								
*Yes*	33	5.86	3.92,7.81	195	8.14	7.05,9.24	228	7.71
*No*	530	94.14	92.19,96.08	2200	91.86	90.76,92.95	2730	92.29
Syphilis								
*Yes*	83	14.74	11.81,17.68	339	14.15	12.76,15.55	422	14.27
*No*	480	85.26	82.32,88.20	2056	85.85	84.45,87.24	2536	85.73

### Behavioral characteristics

More than half of the participants (54.99%) identified themselves as gay men and 38.21% identified themselves as bisexual men. Among the participants, 19.03% (n = 563) engaged in CVI with their female partners, 48.72% (n = 1441) engaged in CAI, more than half (52.40%, 95% CI: 48.26–51.74) of the subjects engaged in CVI were also engaged in CAI. Internet (54.45%) was the preferred way for finding a male partner. About half (48.61%) of the participants had social network size larger than 10(Table 1).

### HIV and syphilis prevalence

The HIV Prevalence among participants engaged in CVI and those who were not were 5.86% (95% CI: 3.92–7.81) and 8.14% (95% CI: 7.05–9.24), respectively. Meanwhile, the syphilis prevalence for the two groups were 14.74% (95% CI: 11.81–17.68) and 14.15% (95% CI: 12.76–15.55), respectively. The HIV and syphilis prevalence among all participants were 7.71% and 14.27%, respectively.

### Bivariate and multivariate analysis

The results of bivariate analysis indicated that compared to participants without any income, participants having income higher than 1000 RMB monthly income were more likely to engage in CVI. With reference to participants aged less than 20 years, participants with age between 40 and 49 had higher likelihood of being engaged in CVI, with crude OR of 3.84 (95% CI: 2.37–6.23).

After adjusting for city, income, race and social network size, multiple logistic regression analysis indicated that older participants were more likely (aOR_40–49years_ = 2.60, 95% CI: 1.54–4.37, and aOR_50 and above_ = 2.25, 95% CI 1.25–4.06) to engage in CVI, compared to participants who were less than 20 years old. Married participants were more likely to engage in CVI (aOR = 6.13, 95% CI: 4.95–7.58), compared to those who were never married. Participants who only attended primary school or below (aOR = 3.86, 95% CI: 2.26–6.69), and attended junior or senior high school (aOR = 2.64, 95% CI 2.10–3.32) were also more likely to engage in CVI, compare to those who attained college or above level of education. Meeting partners in traditional venues such as spa/bathhouse/sauna/massage parlor (aOR = 3.52, 95% CI: 2.63–4.73) or public restroom/public lawn (aOR = 3.50, 95% CI: 2.55–4.82) were associated with CVI, compared to those who met their partners mainly through internet. Participants who identified themselves as heterosexual (aOR = 13.81, 95% CI: 7.14–26.70) or bisexual (aOR = 6.41, 95% CI: 5.05–8.13) had higher likelihood of engaging in CVI, compared to homosexual men. Subjects who were engaged in CAI were also more likely to have exposure to CVI (aOR = 1.29, 95% CI: 1.06–1.57).

The study results further indicated that participants who had correctly answered at least 6 out of the 8 questions regarding transmission and prevention of HIV (aOR = 0.70, 95% CI: 0.55–0.88) and ever received any kinds of HIV related service in the past year (aOR = 0.67, 95% CI: 0.54–0.82) were less likely to engage in CVI.

We also found that subjects who were seropositive for HIV (aOR = 0.85, 95% CI: 0.57–1.27) or syphilis (aOR = 0.99, 95% CI: 0.75–1.30) did not have statistically significant higher likelihood of engaging in CVI, even the observed HIV prevalence was higher among those who were not engaged in CVI in the last six months (Table 2).

**Table 2:** Factors associated with participants who engaged in CVI between 2008 and 2009 in China (N = 2958).

	Crude model			Adjusted model*		
Variables	OR	95% CI	P-Value	OR	95% CI	P-Value
Income						
*No income*	*Ref*					
*1 to 1000*	1.39	0.90,2.13	0.14			
*1001 to 2000*	3.31	2.31,4.73	<0.001			
*2001 to 3000*	3.6	2.47,5.24	<0.001			
*3001 to 4000*	2.89	1.89,4.42	<0.001			
*More than 4000*	2.58	1.62,4.11	<0.001			
City						
*Nanjing*	*Ref*					
*Suzhou*	2.14	1.50,3.06	<0.001			
*Yangzhou*	3	2.13,4.22	<0.001			
*Chongqing*	0.5	0.35,0.72	<0.001			
*Guangzhou*	0.68	0.46,1.01	0.06			
*Ha'erbin*	0.84	0.59,1.21	0.35			
*Jinan*	1.46	1.05,2.02	0.02			
Social network size						
*2 or less*	*Ref*					
*3 to 9*	0.62	0.48,0.80	<0.001			
*10 and above*	0.36	0.28,0.47	<0.001			
Race						
*Han*	*Ref*					
*Others*	0.45	0.21,0.99	0.05			
Age						
*<20*	*Ref*					
*20–29*	0.82	0.52,1.28	0.38	0.69	0.43,1.10	0.12
*30–39*	2.51	1.59,3.97	<0.001	1.83	1.12,2.99	0.02
*40–49*	3.84	2.37,6.23	<0.001	2.6	1.54,4.37	<0.001
*50 and above*	2.23	1.30,3.81	<0.001	2.25	1.25,4.06	0.01
Marital Status						
*Never Married*	*Ref*			*Ref*		
*Ever Married*	7.8	6.39,9.53	<0.001	6.13	4.95,7.58	<0.001
Residency						
*City under study*	*Ref*			*Ref*		
*The province of the city under study*	1.35	1.08,1.69	0.01	0.9	0.70,1.15	0.4
*Other provinces*	1.11	0.89,1.39	0.36	0.84	0.65,1.09	0.19
Education						
*Primary School or below*	5.12	3.05,8.59	<0.001	3.86	2.23,6.69	<0.001
*Junior or Senior High school*	2.91	2.37,3.57	<0.001	2.64	2.10,3.32	<0.001
*College or above*	*Ref*			*Ref*		
Venue						
*Pub*, *Disco*, *Tearoom*, *or Club*	3.16	2.42,4.13	<0.001	2.52	1.89,3.35	<0.001
*Spa or bathhouse*, *Sauna or Massage*	4.73	3.61,6.19	<0.001	3.52	2.63,4.73	<0.001
*Public Restroom*, *or Public Lawn*	3.95	2.97,5.26	<0.001	3.5	2.55,4.82	<0.001
*Internet*	*Ref*			*Ref*		
*Others*	3.03	2.16,4.25	<0.001	2.87	2.02,4.08	<0.001
Sexual Orientation						
*Homosexual*	*Ref*			*Ref*		
*Heterosexual*	16.01	8.52,30.05	<0.001	13.81	7.14,26.70	<0.001
*Bisexual*	7.45	5.92,9.37	<0.001	6.41	5.05,8.13	<0.001
*Not sure*	4.26	2.81,6.45	<0.001	4.09	2.65,6.32	<0.001
Knowledge						
*No*	*Ref*			*Ref*		
*Yes*	0.75	0.60,0.93	0.01	0.7	0.55,0.88	<0.001
Coverage						
*No*	*Ref*			*Ref*		
*Yes*	0.62	0.51,0.75	<0.001	0.67	0.54,0.82	<0.001
CAI						
*No*	*Ref*			*Ref*		
*Yes*	1.2	1.00,1.44	0.05	1.29	1.06,1.57	0.01
HIV						
*Yes*	0.7	0.48,1.03	0.07	0.85	0.57,1.27	0.43
*No*	*Ref*			*Ref*		
Syphilis						
*Yes*	1.05	0.81,1.36	0.72	0.99	0.75,1.30	0.94
*No*	*Ref*			*Ref*		

Note:

* Adjusted for city, income, race and social network size.

## Discussion

Keeping the potential bridging role of bisexual MSM by developing a pathway of transmission of HIV, Syphilis and other STDs between population groups having relatively higher and lower risk of such transmission, current study assessed the burden and associates of CVI among Chinese MSM. In China, MSM contributed to the expanding epidemics of HIV and syphilis not only among their male sexual partners but also to their female sexual partners through condomless sex [3–5, 12, 13]. This multi-centre cross-sectional study tried to add to the literature by using multi-site study design, providing evidence regarding the prevalence of CVI and its associates among MSM in China including socio-economic, demographic and behavioral characteristics along with the prevalence of HIV and syphilis. We also compared MSM who engaged in CVI to other MSM who were not, to understand the potential risk for HIV/syphilis transmission among Chinese MSM and their heterosexual partners.

It was observed that a substantial proportion of the participants were ever or currently married with women and the measured proportion was higher than the overall percentage of marriage with women among Chinese MSM as determined from a recent meta-analysis [14]. Published literature revealed that about 70–90% Chinese MSM might eventually get married owing to cultural expectation and social pressure [11, 15]. Even with this high marriage rate, Chinese MSM were found to rarely disclose their homosexual sexual orientation to their wives [16, 17]. The non-disclosure as well as high risk behaviors of this population with both male and female partners resulted in their potentially crucial in spreading HIV and syphilis between their male sexual partners to their wives or other female sexual partners through condomless sex [14, 18, 19]. This was also the observed scenario in the current study, as almost 10% of the participants were engaged in both CVI and CAI which might have further increased the risk of transmission of HIV and other STDs between the aforementioned groups. The results of this study suggested that the prevalence of HIV was higher among participants compared to the HIV prevalence reported among MSM from the sentinel surveillance program in China [3] and some other localized studies from Beijing[4] and Shandong[5]. This study also observed a high prevalence of syphilis among Chinese MSM, which might have increased the probability of spread of HIV from bisexual MSM to their female sexual partners as syphilis is a known risk factor for HIV transmission [20, 21] However, prevalence of HIV and syphilis were similar between MSM engaging and not engaging in CVI. The observation might be explained by the fact that in China, MSM were observed to have much higher average number of male sexual partners compared to the number of female sexual partners of their bisexual subgroups. Also the risky sexual behavior seemed to be much higher during sexual intercourse with male partners than the female partners. Hence, overall number of instances of risky sexual exposures per MSM and thus risk of transmission of HIV and syphilis seemed to be similar in both groups (those engaging or not engaging in CVI) [3, 20].

According to published literatures, CAI is a well-documented risk factor for HIV/syphilis transmission among MSM [12, 13, 22]. Together with CAI, CVI further increases the risk of spread of HIV/syphilis from MSM to their female sexual partners [3, 5, 14]. Thus, high rates of CAI and CVI together among MSM are likely to increase the risk of HIV and syphilis infection among their female partners. Our study revealed that MSM engaged in CAI were also more likely to engage in CVI. Thus, it seemed that the interaction of CVI and CAI could have intensified the bridging effect for potential transmission of HIV and syphilis between Chinese MSM and their female sexual partners [14, 19, 23].

Our study indicated that participants who mainly found partners from Spa, bathhouse, Sauna or massage parlors were more likely to have CVI. Previously, it was observed that in China, relatively older and less educated MSM usually look for partners from spa, bathhouse, sauna or massage parlors [24]. These MSM were also comparatively less aware of the risk and prevention of HIV and syphilis and related services. Thus they were also more likely to engage in CAI with their male partners [13] and subsequently CVI while having sex with their female partners.

Marriage is an established determinant of CVI for any person. In China, to maintain the social norms, getting married and having children by consummation of marriage are usual expectations towards every single male person. Thus, keeping their other sexual behavior as secret, most of the male persons in this country get married and have sex with women. Considerable proportion of these heterosexual contacts is CVI owing to the principal objective of reproduction. Thus it is obvious that married men compared to their unmarried counterparts are expected to have more CVI even among MSM and this was also observed in prior research [25, 26]. Similarly, participants who self-identified themselves as heterosexuals in the current study also had higher odds of engaging in CVI. Overall, it appeared that the behavioral scenario regarding CVI among MSM in China was critically owing to its potential positive impact on the incidence of HIV and other STDs among female partners of MSM. The situation may also worsen in the near future due to the increasing trend of prevalence of HIV/STDs among MSM and lack of tailored preventive intervention programs targeting these bisexual MSM.

Despite of providing relevant insights, this study had several important limitations, some of which were attributed to the cross-sectional design. First, temporal ambiguity was always a possibility in this study and it was impossible to know the associated factors that were antecedent. Thus we recommend that alike any other observational study these observed associations should not be interpreted as causality. Second, although use of RDS and snowballing could add some representativeness in our study, however, due to the voluntary nature of participation, the lack of generalizability might not be ruled out. Snowballing and RDS could also have introduced some amount of selection bias although we expect the magnitude to be small. Third, as the data were collected through face-to-face interviews, our study might have suffered from the problem of social desirability bias which might have lead to exposure or confounder misclassifications. Fourth, our study did not collect information on the HIV and syphilis status of the female partners of the bisexual MSM, while this information is a key message to measure the bridge effect of these MSM. In addition, even though we adjusted for city, income, race and social network size in the multivariate analysis models, our study might still have residual confounding due to the remaining unknown or unadjusted confounders. Last but not least, the failure to distinguish between partner types–and particularly to distinguish between whether they were in a monogamous ongoing relationship or not is also an important limitation of our study.

## Conclusions

In conclusion, older, married, less educated MSM who usually found their male sex partners from traditional venue like spa, bathhouse, sauna, massage parlor and regarded themselves as heterosexual were more likely to engage in CVI. Results of our study indicated that further intervention should be tailored to this bisexual MSM to prevent spread of HIV, syphilis and other STDs from this group to the general population especially their female partners.

## Supporting Information

S1 Dataset (XLS)
